# Desmoplakin interacts with the coil 1 of different types of intermediate filament proteins and displays high affinity for assembled intermediate filaments

**DOI:** 10.1371/journal.pone.0205038

**Published:** 2018-10-04

**Authors:** Bertrand Favre, Nadja Begré, Jamal-Eddine Bouameur, Prakash Lingasamy, Gloria M. Conover, Lionel Fontao, Luca Borradori

**Affiliations:** 1 Department of Dermatology, Inselspital, Bern University Hospital, Bern, Switzerland; 2 Department for Biomedical Research, University of Bern, Bern, Switzerland; 3 Department of Veterinary Pathobiology, Texas A&M University, College Station, Texas, United States of America; 4 Department of Dermatology, Geneva University Hospitals, Geneva, Switzerland; Medizinische Fakultat der RWTH Aachen, GERMANY

## Abstract

The interaction of intermediate filaments (IFs) with the cell-cell adhesion complexes desmosomes is crucial for cytoskeletal organization and cell resilience in the epidermis and heart. The intracellular desmosomal protein desmoplakin anchors IFs to the cell adhesion complexes predominantly via its four last carboxy-terminal domains (C-terminus). However, it remains unclear why the C-terminus of desmoplakin interacts with different IF types or if there are different binding affinities for each type of IFs that may influence the stability of cell-specific adhesion complexes. By yeast three-hybrid and fluorescence binding assays, we found that the coiled-coil 1 of the conserved central rod domain of the heterodimeric cytokeratins (Ks) 5 and 14 (K5/K14) was required for their interaction with the C-terminus of desmoplakin, while their unique amino head- and C-tail domains were dispensable. Similar findings were obtained *in vitro* with K1/K10, and the type III IF proteins desmin and vimentin. Binding assays testing the C-terminus of desmoplakin with assembled K5/K14 and desmin IFs yielded an apparent affinity in the nM range. Our findings reveal that the same conserved domain of IF proteins binds to the C-terminus of desmoplakin, which may help explain the previously reported broad binding IF-specificity to desmoplakin. Our data suggest that desmoplakin high-affinity binding to diverse IF proteins ensures robust linkages of IF cytoskeleton and desmosomes that maintain the structural integrity of cellular adhesion complexes. In summary, our results give new insights into the molecular basis of the IF-desmosome association.

## Introduction

Desmoplakin is a large desmosomal protein, member of the plakin family of cytolinkers. As an essential component of multiprotein desmosome adhesion complexes, desmoplakin participates in cell-cell adhesion and serves as anchorage sites for intermediate filaments (IFs). Desmosomes are found in all epithelia and some non-epithelial tissues, such as cardiac muscle and meninges [1, 2]. The amino (N)-terminal sequence and the plakin domain of desmoplakin interact with the desmosomal proteins, plakophilin and plakoglobin that associate with the transmembrane cadherins, desmogleins and desmocollins [3, 4], while the central region of desmoplakin forms a parallel coiled-coil dimer [5]. The carboxy (C)-terminal tail of desmoplakin contains three plakin repeat domains (PRDs A, B, and C), a 103 amino acids linker (located after the PRD B), and a carboxyl extremity (E) containing Gly-Ser-Arg (GSR) repeats (S1 Fig) [6]. Previous studies demonstrated that the four C-terminal domains (C-terminus) of desmoplakin are required for its interaction with IFs [7–9]. A similar structural requirement has been observed for the binding of two other plakins to IFs, plectin and the epithelial isoform of bullous pemphigoid antigen 1 (BPAG1e) (S1 Fig) [8, 10, 11]. It is known that desmoplakin anchors different types of IFs to desmosomes, including cytokeratins (Ks) in epithelia, desmin in cardiomyocytes and vimentin in arachnoidal meninges and follicular dendritic cells [7, 12–14]. Phosphorylation of the GSR repeats in the C-extremity of desmoplakin and plectin dynamically regulates the interaction of both proteins with IFs (S1 Fig) [15, 16].

Mutations in the desmoplakin gene (*DSP)* have been associated with human disorders involving tissues exposed to mechanical stress [6, 17]. *DSP* variants often cause cutaneous and cardiac diseases, such as palmoplantar keratoderma, skin fragility-woolly hair syndrome, dilated cardiomyopathy and arrhythmogenic right ventricular cardiomyopathy/dysplasia. Intriguingly, compound heterozygous or homozygous recessive mutations in the *DSP* gene that result in severe truncation of the C-terminus of desmoplakin cause severe skin blistering leading to early death [17]. The critical role of the interaction of desmoplakin with IFs is emphasized by the fact that mutations in either IF or *DSP* genes in human or the knockdown of these genes in mouse models are associated with similar skin or muscle phenotypes [17–19]. Moreover, Ks or desmin knockdown in the epidermis or the heart, respectively, results in mislocalization of desmoplakin [20, 21].

The IF cytoskeletal network imparts resilience to cells and tissues that experience mechanical stress [22, 23]. IF genes are numerous (> 70) and show a broad expression pattern in many cell types. IF proteins display a central α-helical domain of ~310 amino acids (required for dimerization), flanked by globular N- and C-terminal head and tail domains of variable size [24]. The epithelial type I Ks form heterodimeric proteins with their type II partners. Ks are differentially expressed in epithelial cells depending on the specific developmental and differentiation stage of a cell. Vimentin and desmin belong to the type III IF proteins, which also include peripherin and glial fibrillary acidic protein. Notably, the type III IF proteins form both homopolymers and heteropolymers with other type III, IV or VI IF proteins [21].

The assembly process of IFs is initiated by the parallel dimerization of the rod domain and progresses by antiparallel lateral association of the coiled-coils into tetramers that interact with each other via their head domain to form unit length filaments (ULFs). These ULFs subsequently anneal longitudinally and compress to form mature 11 nm-wide IFs [24]. The conserved extremities of the central rod domain are essential for IF assembly, since even subtle amino acid substitutions have devastating effects on IF formation [25, 26]. It is well established that phosphorylation cascades regulate the assembly state of IF networks thereby controlling cell plasticity, cell migration and cell division [27, 28].

Despite the key role of desmoplakin in mediating the anchorage of IFs to desmosomes, the mode of interaction of desmoplakin with IFs is still unclear. Previous studies showed that desmoplakin specifically interacts with the head domain of monomeric type II epidermal Ks (K1, K2, K5, and K6) but not with that of K8 [12]. Both K8 and K18 were necessary to observe an interaction with the desmoplakin tail in yeast two- or three-hybrid (Y2/3H) system [29]. In contrast, we found that desmoplakin preferentially interacts with heterodimers of both epidermal and simple epithelial Ks, independently of their head domain [8, 30]. Thus, this study aims to identify the desmoplakin binding site(s) on IF proteins because this information is critical for understanding the association of IFs with desmoplakin and other plakins and could shed insight into the molecular basis for a number of human disorders affecting the structural integrity of desmosomes. In this study, we have characterized the binding site(s) of the C-terminus of desmoplakin on IF proteins and estimated its apparent affinity for assembled IFs.

## Materials and methods

### Cell culture and transfection

Human epithelial kidney (HEK) 293T cells [31] were cultured in high glucose Dulbecco’s Modified Eagle Medium (Thermo Fischer Scientific), supplemented with 10% fetal calf serum (Sigma-Aldrich), 100 U/mL penicillin and 100 μg/mL streptomycin (Sigma-Aldrich). They were transfected with the calcium phosphate method [32].

### Cloning of cDNA constructs

Plasmids encoding enhanced green fluorescent protein (EGFP) and GAL4-DNA-binding domain (BD) fused to the C-terminus of desmoplakin (DSP C-terminus) (desmoplakin amino acid 2194 to 2871, GenBank accession No: NP_004406), with the amino acid substitution S2849G to prevent inhibitory phosphorylation, were previously described [8]. Human vimentin cDNA cloned in pDS5 was received from H. Herrmann (Heidelberg, Germany). cDNAs encoding full-length or truncated IF proteins were cloned into pAS2.1, pACT2 (Takara Bio Europe) or pACT2-URA [8] for Y2/3H assays, and into pET15b or pET23 (Merck) for expression of recombinant proteins in *Escherichia coli* (S1 Table) [8, 9, 11, 30, 33]. Cloning of mouse K1-coil 1 (C1), mouse K10-C1, human K10-coil 2 to tail (C2-T), human vimentin-rod, -C1, -C2 and human desmin-rod, -C1 and -C2 into pET15b introduced an H_6_-tag at their N-terminus. Plasmids were generated by restriction enzyme-based cloning procedures with or without PCR amplifications. Correctness of PCR-amplified constructs was verified by sequencing.

### Protein sequence alignment

The following IF Genbank protein sequences were aligned with the MAFFT algorithm (version 7.215) [34]; type I Ks: K9, NP_000217; K10, NP_000412; K12, NP_000214; K13, NP_705694; K14, NP_000517; K15, NP_002266; K16, NP_005548; K17, NP_000413; K18, NP_002267; K19, NP_002267; K20, NP_061883; K23, NP_056330; K24, NP_061889; K25, NP_853512; K26, NP_853517; K27, NP_853515; K28, NP_853513; K71, NP_258259; K72, NP_542785; K73, NP_778238; K74, NP_778223; K75, NP_004684; K76, NP_056932; K77, NP_778253; K78, NP_775487; K79, NP_787028; K80, NP_872313; type II Ks: K1, NP_006112; K2, NP_000414; K3, NP_476429; K4, NP_002263; K5, NP_000415; K6A, NP_005545; K6B, NP_005546; K6C, NP_775109; K7, NP_005547; K8, NP_002264; Type III IF proteins: desmin, NP_001918; vimentin, NP_003371.

### Polyacrylamide gel electrophoresis and western blotting

Polyacrylamide gel electrophoresis in presence of sodium dodecyl sulfate (SDS-PAGE) and western blotting (WB) were performed as previously described [8]. Protein concentration was measured with the Bradford protein assay (Bio-Rad) using bovine serum albumin (BSA) (fraction V, AppliChem) as standard and absorbance at 280 nm for purified recombinant proteins.

### Yeast two- and three-hybrid assays

These experiments were performed as previously described [35]. For each pair of Ks or fragments of Ks tested in Y3H, their ability to interact with each other (hetero-dimerization) was verified in Y2H assays with type I and II Ks cloned in pACT2 and pAS2.1, respectively. Growth or no growth on both selection media (without histidine or without adenine) was carefully recorded.

### Expression and purification of recombinant IF proteins

#### K5

Chemicals were from Sigma-Aldrich if not otherwise stated. For K5, 10 mL of Luria-Bertani (LB) medium, supplemented with 0.1% glucose, ampicillin 100 μg/mL and chloramphenicol 34 μg/mL (Amp/Cam), was inoculated with a single colony of *E*. *coli* Rosetta pLysS (Merck), transformed with the plasmid pET23-K5, and incubated at 30°C overnight (ON). The next day, 9 mL of pre-culture was added to 900 mL of LB Amp/Cam + 0.1% glucose and incubated at 37°C until the optical density at 600 nm (OD_600nm_) reaches 0.6. The culture was cooled down in a water bath to room temperature. Expression of K5 was induced by the addition of 0.1 mM isopropyl β-D-1-thiogalactopyranoside (IPTG) and the culture was further incubated at 22°C ON. The next day, the culture was centrifuged at 3000 × *g* at 4°C for 15 min. From the bacterial pellet (4.2 g), inclusion bodies (IBs) were prepared as previously described [11]. IBs were dissolved in the solubilization buffer, 20 mM Bis-Tris-propane, pH 9.0, 6 M urea, 2 mM dithiothreitol (DTT), 100 μM phenylmethylsulfonyl fluoride (PMSF), 10 mL/g wet bacterial pellet. Dissolved IBs were centrifuged at 20,000 × *g* at 4°C for 30 min, dialyzed against the cation exchanger column buffer, 20 mM sodium phosphate, pH 6.5, 6 M urea, 2 mM DTT, 100 μM PMSF, at 4°C ON and centrifuged at 20,000 × *g* at 4°C for 30 min. The supernatant was filtrated through the filter unit Millex GV, 0.22 μm (Merck), and loaded, 20–30 mg total protein/run, onto the cation exchanger column HiTrap SP Sepharose FF 1 mL (GE Healthcare Life Sciences). K5 was eluted with a linear gradient over 30 mL from 0 to 0.375 M NaCl in the cation exchanger column buffer. Pooled fractions were loaded (≤ 0.5 mL/run) on the column Superdex 200 10/300 GL (GE Healthcare Life Sciences) equilibrated with 25 mM Tris-HCl, pH 8.0, 6 M urea, 0.3 M NaCl, 2 mM DTT, 100 μM PMSF. Fractions containing pure K5 were pooled and stored at -80°C (S2 Fig).

#### K14

For K14, 10 mL of LB Amp/Cam + 0.1% glucose, was inoculated with a single colony of *E*. *coli* Rosetta (Merck), transformed with the plasmid pET23-K14, and incubated at 30°C ON. The next day, 9 mL pre-culture was added to 900 mL LB Amp/Cam + 0.1% glucose and incubated 6 h at 37°C (induction of K14 expression with IPTG was dispensable). The culture was centrifuged at 3000 × *g* at 4°C for 15 min. IBs were prepared from bacterial pellet (4.8 g), solubilized and centrifuged as for K5. To the IB solution, 1 M HCl was added dropwise under agitation to decrease the pH from 9.0 to 6.5. The solution was centrifuged at 20,000 × *g* at 4°C for 30 min. The supernatant was filtrated through the filter unit Millex GV, 0.22 μm, and loaded onto the anion exchanger column UNO Q1 (Bio-Rad). K14 was eluted with a gradient over 20 mL from 0 to 0.5 M NaCl in the anion exchanger column buffer, 20 mM Bis-Tris-propane, pH 6.5, 6 M urea, 2 mM DTT, 100 μM PMSF. Pooled samples were gel-filtrated as for K5 (S2 Fig).

#### K1, K8, K10, K18 and H_6_-tagged K fragments

K8 and K18 as well as K1, H_6_-K10 and H_6_-tagged K fragments were expressed and purified as previously described [30]. H_6_-K10 was further gel-filtrated in the same conditions as for K5 (S2 Fig).

#### Vimentin and desmin

Vimentin was purified following a published protocol [36]. LB Amp/Cam + 0.1% glucose, 15 mL, was inoculated with a colony of *E*. *coli* Rosetta (DE3), transformed with the plasmid pDS5-vimentin, and incubated at 30°C ON. To 1.3 L LB Amp/Cam + 0.1% glucose, 13 mL of pre-culture was added and incubated at 37°C until OD_600nm_ = 0.6. Vimentin expression was induced with 0.1 mM IPTG at 37°C for 5 h. IBs were prepared from bacterial pellet (10.5 g) as for K5. They were dissolved in 50 mL of the anion exchanger column buffer, 5 mM Tris-HCl, pH 7.5, 8 M urea, 1 mM DTT, 100 μM PMSF, 1 mM EDTA, and 0.1 mM EGTA, and centrifuged at 20,000 × *g* at 4°C for 30 min. Supernatant was filtrated through the filter unit, Millex GV, 0.22 μm, and loaded onto the anion exchanger column HiTrap DEAE 1 mL (GE Healthcare Life Sciences), 30 to 40 mg protein/run. Vimentin was eluted with a linear gradient from 0 to 0.3 M KCl in the anion exchanger column buffer. Pooled fractions were dialyzed against the previous anion exchanger column buffer and loaded, 15 mg protein/run, onto the cation exchanger column HiTrap CM Sepharose, 1 mL (GE Healthcare Life Sciences), equilibrated with the anion exchanger column buffer. Vimentin was eluted with a linear gradient over 20 mL from 0 to 0.3 M KCl in the anion exchanger column buffer. Pooled samples were gel filtrated as for K5. Pure samples were supplemented with 10 mM methyl ammonium chloride and stored at -80°C. Similarly, desmin was purified following a published protocol [37].

Vimentin-rod, -coil 1, -coil 2, desmin-rod, -coil 1 and -coil 2 were expressed in *E*. *coli* BL21 (DE3) as for vimentin. After centrifugation of the cultures at 3000 × *g* at 4°C for 15 min, bacterial pellets were directly solubilized in SDS-sample buffer, 1/10 of OD_600nm_ × culture volume (mL).

#### SUMO

For H_6_-small ubiquitin-related modifier (SUMO), LB Amp/Cam + 0.1% glucose (10 mL) was inoculated with *E*. *coli* Rosetta transformed with pET-SUMO (Thermo Fischer Scientific) and incubated at 37°C ON. A 4 mL-fraction of the preculture was added to 400 mL LB Amp/Cam + 0.1% glucose that was incubated at 37°C until OD_600nm_ = 0.6. Expression of SUMO was induced by the addition of 0.1 mM IPTG. The culture was further incubated at 37°C for 2.5 h and centrifuged at 3000 × *g* for 15 min at 4°C. The pellet was resuspended in 10 mL of lysis buffer 20 mM sodium phosphate, pH 7.4, 0.5 M NaCl, 20 mM imidazole, 1/100 protease inhibitor cocktail set III (Calbiochem). The bacteria in suspension were lysed with several 30 s-long sonication pulses using the apparatus Labsonic 2000 (Braun). Lysates were centrifuged at 20,000 × *g* at 4°C. The supernatant was filtrated through the filter Millex GV, 0.22 uM, and loaded onto the column HisTrap 1 mL (GE Healthcare). H_6_-SUMO was eluted with the lysis buffer supplemented with 0.5 M imidazole, dialyzed against PBS, flash frozen in liquid nitrogen and kept at -80°C.

### FluoBACE interaction assays

#### Spotting conditions for dot blots

IF proteins were spotted in the following buffers: K5/K14 and BSA in 25 mM Tris–HCl, pH 7.4, 2 mM DTT, 8 M urea; K1/K10 in 25 mM Tris–HCl, pH 7.4, 2 mM DTT, 2 M urea; K8/K18 in 10 mM Tris–HCl, pH 8.5, 2 mM DTT, 6 M urea; vimentin in 5 mM Tris-HCl, pH 8.5, 1 mM DTT, 6 M urea, 1 mM EDTA, 0.1 mM EGTA; desmin in 5 mM Tris-HCl, pH 8.5, 1 mM DTT, 4 M urea, 1 mM EDTA, 0.1 mM EGTA. Fragments of K5/K14 and K1/K10 were spotted in the same conditions as full-length proteins [38–41]. When Ks, full-length or fragment proteins, were mixed, they were considered as dimeric entities to calculate the amount of proteins to be spotted to have the same number of moles as the individual monomeric proteins.

#### Interaction of K5 with K14-C2-T in spotting conditions used for dot blots

Purified K5 and H_6_-K14-C2-T were dialyzed against 25 mM Tris–HCl, 2 mM DTT, 8 M urea, pH 7.4 and either loaded separately (6 nmol in 0.25 mL) or after being stoichiometrically mixed onto the column Superdex 200 10/300 GL equilibrated in the same buffer. Eluted fractions were analyzed by SDS-PAGE. Well separated individual elution peaks (retention time of K5 was higher than that of H_6_-K14-C2-T) merged into an early elution peak (retention time shorter than that of H_6_-K14-C2-T) when both proteins were mixed together before the run.

#### Fluorescence overlay assays

Dot blot overlay assays were done as previously described [11, 30]. For overlay of WBs, prepared membranes were stained with Ponceau-S, photographed with a digital camera (FusionPulse TS, Vilber Lourmat), washed several times in PBS, blocked in PBS supplemented with 5% skim milk for 30 min at 4°C, incubated with the soluble fraction of HEK 293T cells expressing EGFP or EGFP-tagged proteins for 1 h at 4°C, washed 3 × with PBS for 5 min at 4°C and scanned for fluorescence with a Typhoon 9400 (GE Healthcare Life Sciences). Fluorescence and Ponceau-S signals were quantified with ImageQuant (GE Healthcare Life Siences) and Image Studio (Li-COR), respectively. Results were plotted with GraphPad Prism 7 (GraphPad Software, Inc.) and statistically analyzed using the one-way ANOVA test, no matching-pairing, assumed Gaussian distribution, followed by the Tukey multiple comparison test (ANOVA-Tukey).

#### Assembly of IFs from recombinant proteins

K5/K14 filaments were assembled as previously described [42]. Pure K5 and K14 were mixed at a 1.1:1 molar ratio, dialyzed against the anion exchanger column buffer, 50 mM Tris-HCl, pH 8.0, 8 M urea, 2 mM DTT, 100 μM PMSF, and loaded onto the anion exchanger UNO Q1 column, 1 mL. Elution was performed with a gradient over 25 mL from 0 to 0.25 M NaCl in the anion exchanger column buffer (heterodimeric K5/K14 elutes last). K5/K14 heterodimers, 0.5 mg/mL, were dialyzed against 0.7 mM sodium phosphate, pH 7.5 (mixture of mono- and di-basic sodium phosphate, 0.7 mM each, to reach the correct pH), 1 mM DTT. Protein concentration was re-measured after dialysis. IF assembly was induced by the addition under agitation of 1/5 of volume of 0.7 mM sodium phosphate, 120 mM KCl, pH 7.5 (20 mM KCl final) and incubation at 37°C for 30 min. Formation of insoluble filaments was verified by centrifugation of aliquots (30 μL, 25 μg/mL) at 20,000 × *g* at 4°C for 30 min and analysis of proteins in the pellet and supernatant by SDS-PAGE. K5/K14 was ≥ 90% insoluble.

Desmin, 0.6 mg/mL, was dialyzed against 2 mM sodium phosphate pH 7.5 (mixture of mono- and di-basic sodium phosphate, 2 mM each, to reach the correct pH). After dialysis, the concentration of desmin was determined. IF assembly was induced by the addition under agitation of one volume (1:1) of 2 mM sodium phosphate pH 7.5, 200 mM KCl (100 mM KCl final) [43] and incubation at 22°C for 1 h. Formation of insoluble filaments was checked by centrifugation of aliquots (30 μL, 25 μg/mL) at 20,000 × *g* at 22°C for 30 min. Analysis of proteins in the pellet and supernatant by SDS-PAGE revealed that desmin was ≥ 90% insoluble.

For each binding assay, the whole assembly procedure, consisting of IF protein(s) dialysis, IF-assembly induction in the high ionic strength buffer and final dilution(s), was performed just before use.

#### Fluorescence binding assays to assembled IFs

To prepare cell lysates used in our binding assays on *in vitro* assembled IFs, HEK 293T cells, expressing EGFP-DSP C-terminus or EGFP, were lysed in the same buffer used to assemble K5/K14 or desmin IFs (see above), supplemented with 0.1% Triton X-100 and 1/100 protease inhibitor cocktail (Sigma-Aldrich). Cell lysates were centrifuged at 20,000 × *g* at 4°C for 30 min and the supernatants saved. Both the protein concentration and fluorescence were measured in these soluble fractions. Soluble extracts from non-transfected cells, diluted at the same protein concentration as that of fluorescent soluble extracts with the appropriate IF-assembly buffer, were used to prepare serial dilutions of fluorescent cell extracts so that the final protein concentration was the same in all fluorescent samples. Serially diluted cell extracts containing EGFP-DSP C-terminus or EGFP, 40 μL each, were mixed with 10 μL of *in vitro* assembled IFs, 125 μg/mL (25 μg/mL final), incubated 15 min either at 4°C with K5/K14 IFs or at 22°C for desmin under agitation (300 rotation per minute) and centrifuged at 20,000 × *g* at 4°C or 22°C, respectively, for 30 min. Fluorescence in the supernatant, 5 μL sample to 95 μL PBS, 0.1% BSA in 96-well plate with round bottom in triplicate, was measured with Tecan Spark 10M (Tecan). EGFP-DSP C-terminus and EGFP concentrations were determined using a fluorescence standard curve obtained with pure recombinant EGFP-H_6_ [30]. Binding curves were fitted with GraphPad Prism 7, non-linear regression, one site, specific binding with Hill slope. SDS-PAGE analysis of the insoluble fractions of the binding assays, previously washed with 40 μL assembly buffer, indicated that the insolubility of K5/K14 IFs or desmin IFs was not affected by the addition of the soluble fraction of HEK 293T cells.

To measure the percentage of active IF-binding EGFP-DSP C-terminus among the soluble extracts of transfected HEK 293T cells, the concentration of EGFP-DSP C-terminus was kept constant at 25 nM whereas that of *in vitro* assembled K5/K14 or desmin IFs was varied from 0 to ≥ 250 μg/mL in a final volume of 50 μL. At the highest concentration of IFs, insoluble fluorescence was ≥ 90% of the fitted Bmax value. Remaining soluble fluorescence (% of total fluorescence) was considered as inactive EGFP-DSP C-terminus [44].

## Results

### The C-terminus of desmoplakin interacts with the coil 1 of epidermal Ks, desmin, and vimentin

Previous studies, using Y3H assays and overlay assays, showed that the tail and C-terminus of desmoplakin preferentially bound to heterodimeric Ks K1/K10, K5/K14, and K8/K18 when compared with monomeric Ks [8, 30]. Similar results were obtained in quantitative fluorescence-based overlay assays with the C-terminus of desmoplakin tagged at the N-terminus with EGFP (EGFP-DSP C-terminus) (S3 Fig). Together, these results indicate that the quaternary structure associated with dimerization/tetramerization of Ks is a key factor for their association with the tail and C-terminus of desmoplakin.

**Fig 1:**
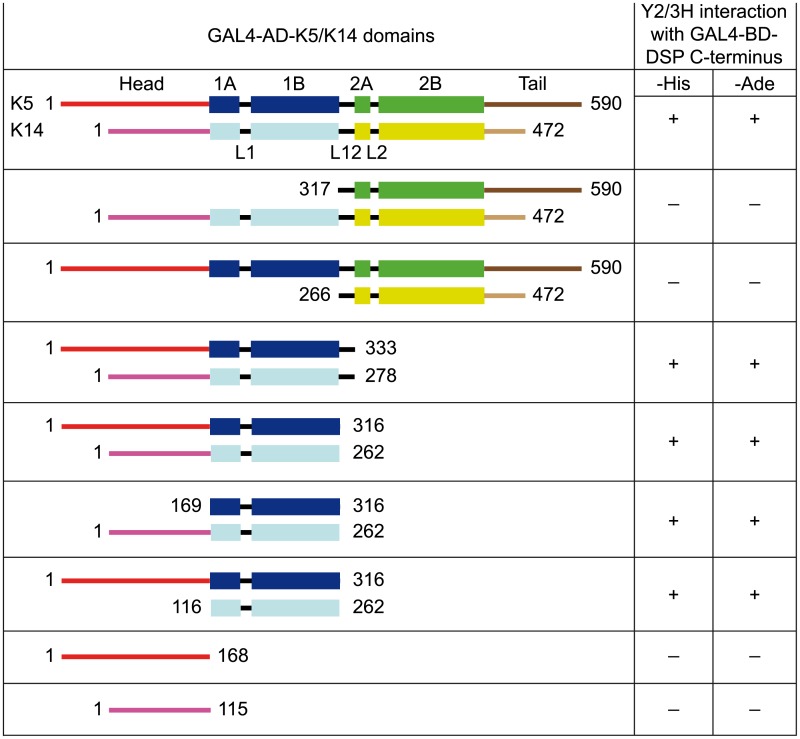
The coil 1 of K5/K14 is essential for their interaction with the C-terminus of desmoplakin in Y3H assays. Summary of Y2/3H results obtained with the indicated constructs. Numbers in each protein show amino acid order. K domains are labeled; the coils are drawn as boxes. + or − means growth or no growth, respectively, on selection medium without histidine (His) or adenine (Ade).

**Fig 2:**
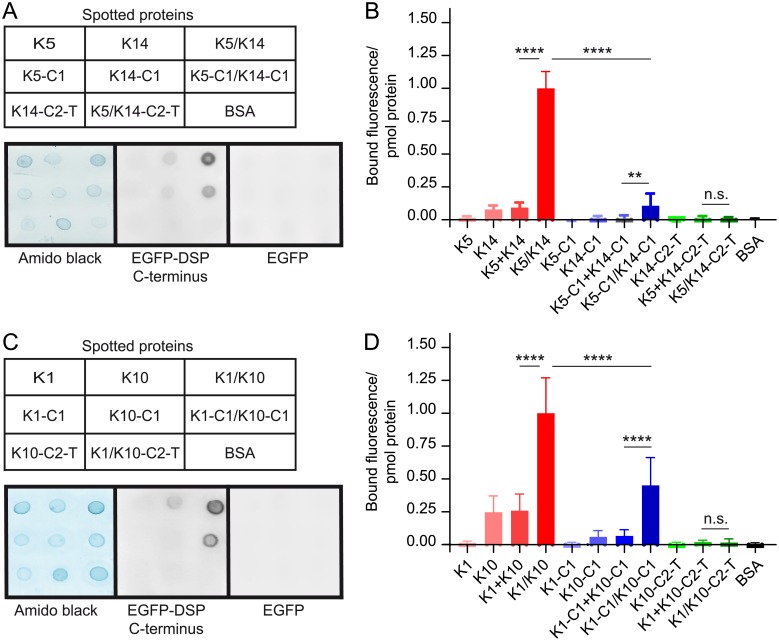
The coil 1 of K5/K14 and K1/K10 contains the major binding site(s) for the C-terminus of desmoplakin in overlay assays. (A) Nitrocellulose membranes, spotted as indicated with bovine serum albumin (BSA, control) and individual or mixed (/) K5 and K14, either full-length proteins or the fragments coil 1 (C1) and coil 2 to tail (C2-T) (3 pmol/spot), were stained with amido black or overlaid with soluble extracts of HEK 293T cells expressing either EGFP-DSP C-terminus (64 ± 29 nM, total concentration) or EGFP (≥ 70 nM) and scanned for fluorescence. (B) Quantified fluorescence signals are relative to the normalized results obtained with the mixture (/) of full-length K5/K14. K5 + K14 and K5-C1 + K14-C1 correspond to the sum of fluorescence signals obtained with the individual proteins (not mixed). Mean ± SD, n = 3; ANOVA-Tukey test; ** and ****, residual *P* < 0.01 and < 0.0001, respectively. (C) and (D) as for (A) and (B) with K1 and K10.

To dissect sequences within IF proteins essential for their binding to desmoplakin in Y3H assays, we generated a series of deletion constructs encompassing various portions of K5 and K14. First, we showed that truncated K5 and K14 proteins were still able to interact with each other (heterodimerization) in Y2H assays like the full-length proteins (S2 Table). Next, we tested whether truncated K5 and K14 could interact with the C-terminus of desmoplakin in Y3H assays. Our analyses revealed that the co-expression of K5 and K14 proteins containing their coil 1 was indispensable for interaction with the C-terminus of desmoplakin (Fig 1). Our findings were validated in follow-up FluoBACE assays. EGFP-DSP C-terminus bound to the pair consisting of H_6_-tagged coils 1 of K5 and K14. However, it did not interact with the pair made of full-length K5 and H_6_-tagged coil 2 to tail (C2-T) of K14 (Fig 2A and 2B). Identical results were obtained when deletion mutants of K1 and K10, corresponding to those of K5 and K14, were tested in overlay assays (Fig 2C and 2D). Hence, our results strongly suggest that the heterodimeric coil 1 of epidermal Ks is a major binding site for the C-terminal region of desmoplakin. Nevertheless, since in our assays the binding of the C-terminus of desmoplakin to K-coils 1 was systematically weaker than to the full-length proteins (Fig 2B and 2D), we cannot exclude the possibility that there are additional binding sites on Ks contributing to their interaction with desmoplakin.

**Fig 3:**
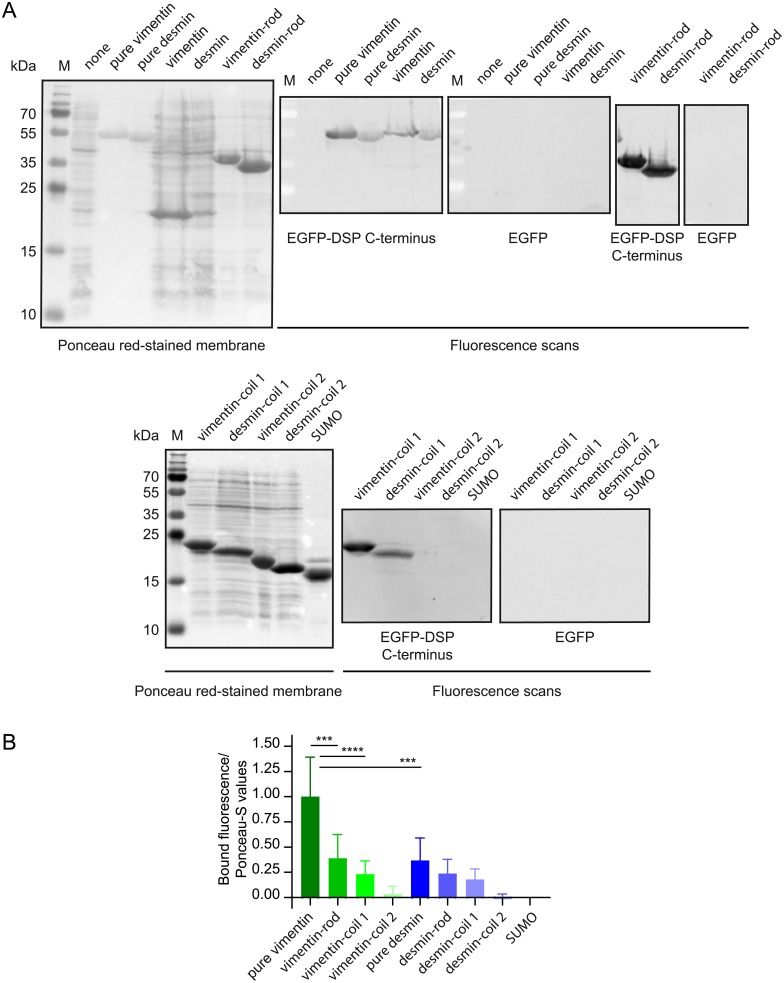
The C-terminus of desmoplakin binds to the coil 1 of desmin and vimentin. (A) Purified vimentin, desmin and SUMO (negative control) (4 μg/lane), or whole extracts from *E*. *coli* expressing full-length vimentin or desmin, the rod, coil 1 and coil 2 of these proteins or no exogenous protein (none), as indicated, were size-fractionated on 15% SDS-PAGE and transferred to WB membranes that were stained with Ponceau-S and overlaid with soluble extracts from HEK 293T cells expressing EGFP-DSP C-terminus or EGFP (both at 67 ± 17 nM, total concentration). M, markers. (B) Quantified fluorescence signals are relative to the normalized results obtained with full-length vimentin, mean ± SD, n≥4; ANOVA-Tukey test; *** and ****, residual *P* < 0.001 and < 0.0001, respectively.

Based on our previous study that shows that the C-terminus of desmoplakin interacts *in vitro* with tail-less type III IF proteins [9], we decided to also characterize the IF-binding site(s) on vimentin and desmin by the FluoBACE method. These IF proteins were expressed in *E*. *coli* and transferred by electrophoresis to a nitrocellulose membrane after size-fractionation on denaturing polyacrylamide gels (SDS-PAGE). EGFP-DSP C-terminus consistently bound to full-length desmin and vimentin, their rod and coil 1 domains but not to their coil 2 domain (Fig 3A). Interestingly, it was previously reported that both the vimentin coil 1 and 2 homodimerize *in vitro* to similar extents [45]. Despite the absence of binding of the C-terminus of desmoplakin to the coil 2 of vimentin or desmin and to SUMO, analysis of results with one-way ANOVA-Tukey test indicates that only the binding of the C-terminus of desmoplakin to full-length vimentin is significantly different from all the other proteins (Fig 3B). In contrast, a *t*-test between the coils 1 and 2 of vimentin or desmin gives *P* values ≤ 0.01. These results imply that the coil 1 domain of both dimeric types I/II as well as of III IFs contains a conserved motif that serves as a major binding site for the C-terminus of desmoplakin.

Next, we analyzed the impact of selected amino acid substitutions associated with epidermolysis bullosa simplex (EBS) located within or close to the coil 1 of either K5 or K14 on the ability of the heterodimeric proteins to interact with the C-terminus of desmoplakin in Y3H assays. None of the tested EBS variants, K5 p.(Glu168Asp), p.(Glu168Gln), p.(Asn176Ser), p.(Asn177Ser), p.(Leu311Arg) and K14 p.(Arg125His), p.(Ile176Met), p.(Val270Met), p.(Leu284Pro), prevented the interaction with the C-terminus of desmoplakin when associated with its wild-type K partner (S3 Table). Consequently, impaired binding of these EBS K mutants to desmoplakin does not seem be to be a major factor for their pathogenicity.

### The C-terminus of desmoplakin has high affinity binding sites for K5/K14 and desmin IFs

**Fig 4:**
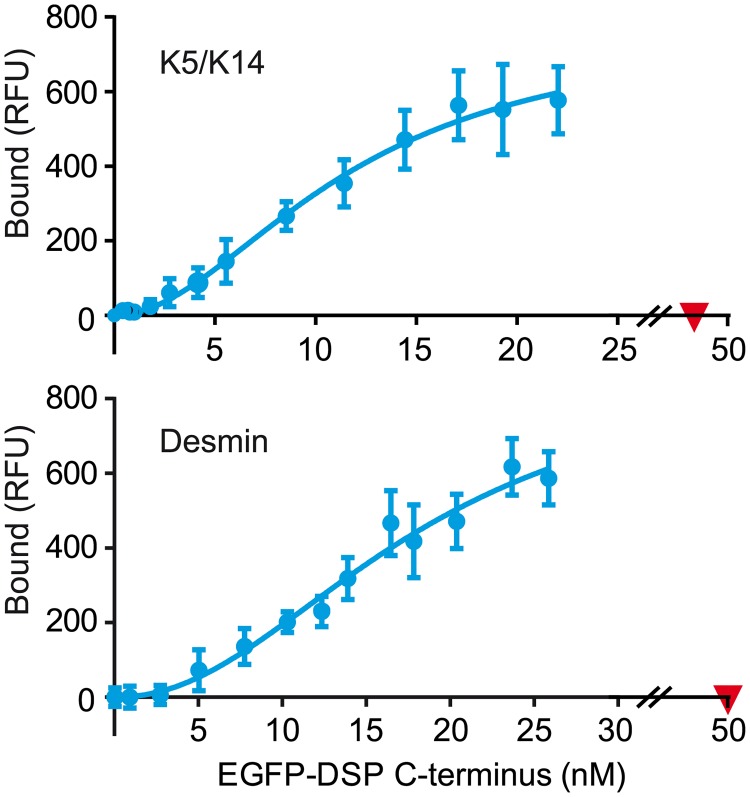
Binding of the C-terminus of desmoplakin to K5/K14 and desmin IFs under equilibrium condition. Variable concentrations of active EGFP-DSP C-terminus (blue, dots) in soluble HEK 293T cell extracts were mixed with a constant concentration of *in vitro* assembled IFs (25 μg/mL). Binding of the control EGFP (red, inverted triangles) was measured at the highest concentration of total (active + inactive) EGFP-DSP C-terminus. Representative binding curves from three independent experiments are shown (RFU, relative fluorescence unit).

Several studies have provided experimental evidence that the C-terminus of desmoplakin contains different domains that synergistically contribute to robust binding to IFs and confer mechanical resilience to cells [7–9, 29, 46, 47]. Nevertheless, the apparent affinity of the C-terminus of desmoplakin for IFs has not been determined. To this end, we used our FluoBACE assay on K5/K14 and desmin IFs assembled *in vitro* [42, 43]. First, we measured the fraction of active EGFP-DSP C-terminus in the soluble fraction of HEK 293T cells by keeping the concentration of EGFP-DSP C-terminus constant and increasing the amount of IFs (S4 Fig). About 50% (47 ± 12%, ± SD, n = 7) of the total fluorescence was bound to IFs formed either by K5/K14 or desmin. It is possible that improper folding or inhibitory posttranslational modifications account for the inability of the remaining expressed portion of EGFP-DSP C-terminus to interact with IFs. Next, we varied the concentration of EGFP-DSP C-terminus present in soluble HEK 293T cell extracts and kept that of K5/K14 or desmin IFs constant (25 μg/mL). Despite the limitations of this approach, due to the maximal available concentration of active protein (≤ 30 nM), we found an apparent Kd of 10 ± 2 nM for K5/K14 IFs and 18 ± 3 nM for desmin IFs with a positive cooperativity (Hill coefficient, h) of 2.4 ± 0.6 or 2.3 ± 0.2 (mean ± SD, n = 3), respectively (Fig 4). The control EGFP did not bind to insoluble IFs. Unfortunately, it is technically challenging to produce intact soluble EGFP-DSP C-terminus in *E*. *coli* to test higher concentrations [30]. Since IF proteins require specific assembly conditions in high ionic strength buffers, the apparent Kd values obtained with the C-terminus of desmoplakin for K5/K14 and desmin cannot be directly compared.

## Discussion

In this study, we describe the binding properties of desmoplakin to epidermal Ks and type III IF proteins desmin and vimentin using complementary biochemical binding assays. Our data provide for the first time evidence that the coil 1 of heteromeric K5/K14, K1/K10 and homomeric desmin and vimentin IF proteins contain recognition sites that bind desmoplakin. Our finding contradicts the reported specificity of desmoplakin and its tail for the unique head domain of most type II Ks except K8 [12, 29]. Specifically, Kouklis et al. [12] reported that a region encompassing the KSIS motif within the head domain of K1 and K5 was crucial for the interaction of desmoplakin with epidermal Ks. Similarly, Meng et al. [29] found that the head domain of K1 was essential for its interaction with desmoplakin by Y2H assays and by peptide competition in *in vitro* binding experiments. Furthermore, no association of the tail of desmoplakin with vimentin could be demonstrated in immune overlay assays [11]. The discrepancies between these results and ours may be due to the use in overlay assays of improperly folded desmoplakin proteins that were purified in denaturing conditions [12, 29]. In contrast to the latter studies, here and in our previous investigations we used native recombinant desmoplakin proteins [8, 9].

Depending on the assay, Y2H or overlay with *in vitro* translated proteins, we found that the tail of desmin was either critical or dispensable, respectively, for its interaction with desmoplakin [14]. It is worth noting, however, that Y2H system is a screening strategy that is prone to false positive and negative results that must be verified with additional tests [47]. Here, we used a second binding assay that confirms that the tails of desmin and vimentin are dispensable for their interaction with the C-terminus of desmoplakin [9]. This finding is supported by the fact that the tails of IF proteins significantly differ from each other and are thus unlikely to provide a common binding site that accounts for the broad specificity of desmoplakin for IFs. The same remark applies to the head domains of IF proteins.

Our binding results, describing desmoplakin and IFs association, agree well with those reported for plectin, another plakin protein with a similar C-terminal structure (S1 Fig). The C-terminus of plectin predominantly binds to the coil 1 domain of K5/K14, desmin, and vimentin [11, 30, 33]. Furthermore, the unique PRD of envoplakin interacts with the monomeric coil 1 of vimentin [48]. Based on these results, it is tempting to conclude that plakins bind to a similar set of sequences within the conserved dimeric/tetrameric coil 1 region of Ks and type III IF proteins (S5 Fig). The interaction of the PRD B of envoplakin with the monomeric coil 1 of vimentin seems to depend on ionic interactions between basic amino acids in the former (Lys1901 and Arg1914) and acidic amino acids in the coil 1A of the latter (Asp112 or Asp119) [48]. However, salt concentrations did not affect the interaction of desmoplakin PRDs with vimentin [46]. Therefore, the exact contribution of ionic interactions between the C-terminus of desmoplakin and IF proteins remains to be determined in future studies.

One of the most common causes for EBS are heterozygous missense variants in the genes encoding K5 and K14, *KRT5* and *KRT14* respectively. These mutations, which are typically found in conserved helix boundary motifs of the rod domain, are thought to be pathogenic by affecting the assembly of K5/K14 in keratinocytes, although additional mechanisms could be involved [24, 49, 50]. We therefore tested the hypothesis that defective linkage of IFs to cell membrane sites could contribute to the cytoskeletal disorganization observed in EBS keratinocytes. However, despite the crucial binding of desmoplakin to the coil 1 of Ks, none of the analyzed EBS-associated mutations within or close to the coil 1 of K5 or K14 prevented the interaction with desmoplakin in Y3H assay. Nevertheless, because of the mainly qualitative properties of the Y2/3H system (growth/no growth), it cannot be excluded that some of these mutations weakened the interaction with desmoplakin but not enough to abolish it, even on the medium without adenine, which is more stringent than the medium without histidine. Recently, we reported that several amino acid substitutions in the PRD B and C of the C-terminus of desmoplakin reduce its interaction with various Ks and type III IF proteins [51, 52]. This indicates a common binding mode of the C-terminus of desmoplakin to different IFs.

The interaction of desmoplakin with the coil 1 of IF proteins provides a logical explanation for the broad specificity of most plakins for various IF proteins. However, it should be emphasized that the C-terminus of desmoplakin bound more strongly to full-length IF proteins than to their coil 1. This observation may be due to either variable accessibility to the binding sites on smaller proteins immobilized on a nitrocellulose membrane or to the different assembly stage of immobilized full-length proteins versus coil 1 dimers. It cannot be excluded either that domains other than the coil 1 within IF proteins synergistically contribute to the binding of IFs to desmoplakin.

Based on the obtained FluoBACE binding curves the apparent Kd of the C-terminus of desmoplakin for IFs is in the nM range, which is comparable to that of plectin (S4 Table). This high binding affinity probably constitutes a requisite for the robust anchoring of IFs to membrane sites to impart mechanical resilience *in vivo*. Actually, the affinity of the tail of desmoplakin for IFs could be even higher than that of the C-terminus because there are reports indicating that the PRD A is also involved in the binding of desmoplakin to IFs [46, 53]. However, the lower expression level of the tail compared to the C-terminus of desmoplakin in HEK 293T cells and our inability to produce intact soluble tail of desmoplakin in *E*. *coli* [30] have prevented us to measure the affinity for IFs of the entire tail of desmoplakin, comprising the PRD A domain.

Although the association between IFs and the C-terminus of desmoplakin is strong, there are regulatory mechanisms that temporarily modulate this interaction. In fact, posttranslational modifications, such as phosphorylation events, control not only the assembly stage of IFs but also the interaction between desmoplakin and IFs in processes such as cell migration, cell mitosis and stress responses [28, 54]. Both desmoplakin and plectin contain two clusters of phospho-sites flanking their last PRD (S1 Fig). However, while the C-terminal cluster of phospho-sites has been partially characterized [15, 16], nothing is known about the role of the phospho-sites located before the last PRD.

In conclusion, the interaction of desmoplakin with Ks and type III IF proteins mainly depends on the conserved coil 1 domain of IF proteins, an observation explaining the broad specificity of desmoplakin for various IFs.

## Supporting information

S1 Fig Domain organization of the tail and C-terminus of desmoplakin; comparison with the C-termini of plectin and BPAG1e; sequence alignment of two clusters of phosphorylated sites located in the C-termini of desmoplakin and plectin. A) Amino acid labeling of desmoplakin (DSP), plectin (PLEC) and BPAG1e corresponds to GenBank protein sequences NP_004406, NP_958782 and NP_001714, respectively; carboxyl extremity, E. B) P-cluster 1 precedes the last PRD in both proteins, PRD 3/C in DSP and PRD 6/C in PLEC. The first amino acid of these PRDs is in blue. Phosphorylated residues are in red, the underlined residues were found phosphorylated ≥ 10 × for plectin and ≥ 5 × for DSP (www.phosphosite.org). C) P-cluster 2 is located in the COOH-extremity of both proteins just after the last PRD C. The last amino acid of the PRD C is in blue. Methylated Arg are in green. Identified protein kinases and phosphatase are indicated. GSK3β, glycogen synthase kinase 3β; MNK2, mitogen-activated protein kinase-interacting serine/threonine protein kinase 2; PKA, cyclic AMP-dependent protein kinase; PP2A, protein phosphatase 2A.
(PDF)

S2 Fig SDS-PAGE analysis of purified recombinant Ks. The indicated proteins (1 μg/lane) were size fractionated on 12% SDS-PAGE that was stained with Coomassie brilliant blue. M, markers.
(PDF)

S3 Fig The C-terminus of desmoplakin binds more strongly to heteromeric than monomeric Ks in fluorescence overlay assays. A) Nitrocellulose membranes, spotted with BSA (control) and individual or mixed (/) cytokeratins (3 pmol/spot) were stained with amido black or overlaid with soluble extracts of HEK 293T cells expressing either EGFP-DSP C-terminus (55 ± 21 nM, total concentration) or EGFP (≥ 60 nM) and scanned for fluorescence. B) Quantified fluorescence signals are relative to the normalized results obtained with each mixture (/) of keratins (K1/K10, K5/K14 and K8/K18). K1 + K10, K5 + K14 and K8 + K18 correspond to the sum of the fluorescence signals obtained with the individual proteins (not mixed). Mean ± SD, n≥3; ANOVA-Tukey test; ** and ****, residual *P* < 0.01 and < 0.0001, respectively.
(PDF)

S4 Fig Saturation binding curves of K5/K14 and desmin IFs to the C-terminus of desmoplakin under equilibrium conditions. Variable concentrations of K5/K14 IFs or vimentin IFs, preassembled *in vitro*, were mixed with a constant concentration (22 nM) of EGFP-DSP C-terminus or EGFP in the soluble extracts of HEK 293T cells. Representative binding curves from ≥ 3 independent experiments are shown.
(PDF)

S5 Fig Sequence alignment of the coil 1 of Ks, desmin and vimentin. The MAFFT algorithm was used; *,:, and. denote identity, conserved and semi-conserved amino acid substitutions, respectively. The position in the protein sequence of the first amino acid in each row is indicated.
(PDF)

S1 Table Tested recombinant IF proteins. (PDF)

S2 Table Y2H assay results with some K5 and K14 constructs used to map the interacting domain(s) with the C-terminus of desmoplakin in Y3H assays. (PDF)

S3 Table *KRT5* and *KRT14* variants associated with EBS and tested in Y3H assays with their wild-type partner and the C-terminus of desmoplakin. (PDF)

S4 Table Apparent Kd of plakins for IF proteins reported in the literature. (PDF)
