# Supplementary material for: Desmoplakin interacts with the coil 1 of different types of intermediate filament proteins and displays high affinity for assembled intermediate filaments
Source: PLoS One. 2018 Oct 4;13(10):e0205038. doi: 10.1371/journal.pone.0205038 (PMC6171917; doi:10.1371/journal.pone.0205038)
Supplement: S1 Fig — A) Amino acid labeling of desmoplakin (DSP), plectin (PLEC) and BPAG1e corresponds to GenBank protein sequences NP_004406, NP_958782 and NP_001714, respectively; carboxyl extremity, E. B) P-cluster 1 precedes the last PRD in both proteins, PRD 3/C in DSP and PRD 6/C in PLEC. The first amino acid of these PRDs is in blue. Phosphorylated residues are in red, the underlined residues were found phosphorylated ≥ 10 × for plectin and ≥ 5 × for DSP (www.phosphosite.org). C) P-cluster 2 is located in the COOH-extremity of both proteins just after the last PRD C. The last amino acid of the PRD C is in blue. Methylated Arg are in green. Identified protein kinases and phosphatase are indicated. GSK3β, glycogen synthase kinase 3β; MNK2, mitogen-activated protein kinase-interacting serine/threonine protein kinase 2; PKA, cyclic AMP-dependent protein kinase; PP2A, protein phosphatase 2A. (PDF) [file pone.0205038.s001.pdf]

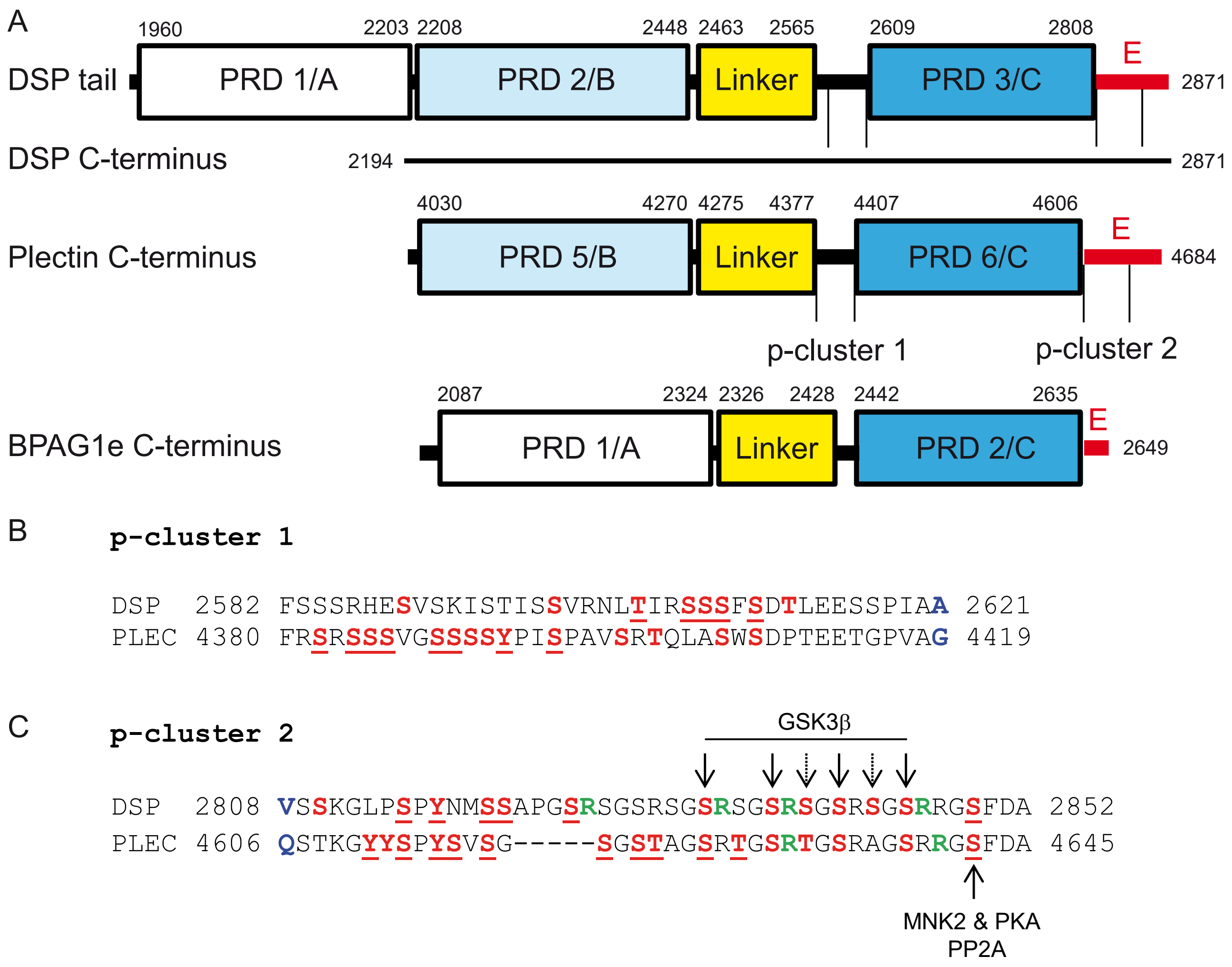
A
DSP tail
1960
PRD 1/A
2203 2208
PRD 2/B
2448 2463
Linker
2565
2609
PRD 3/C
2808
E
2871
DSP C-terminus
2194
2871
Plectin C-terminus
4030
PRD 5/B
4270 4275
Linker
4377
4407
PRD 6/C
4606
E
4684
p-cluster 1
p-cluster 2
BPAG1e C-terminus
2087
PRD 1/A
2324 2326
Linker
2428
2442
PRD 2/C
2635
E
2649
B
p-cluster 1
DSP 2582 FSSSRHESVSKISTISSVRNLTIRSSSFSDTLEESSPIAA 2621
PLEC 4380 FRSRSSSVGSSSSYPISPAVSRTQLASWSDPTEETGPVAG 4419
C
p-cluster 2
GSK3β
DSP 2808 VSSKGLPSPYNMSSAPGSRSGSRSGSRSGSRSGSRSGSRRGSFDA 2852
PLEC 4606 QSTKGYYSPYSVSG-----SGSTAGSRTGSRTGSRAGSRRGSFDA 4645
MNK2 & PKA
PP2A
