# Supplementary figures and images for: Desmoplakin interacts with the coil 1 of different types of intermediate filament proteins and displays high affinity for assembled intermediate filaments
Source: PLoS One. 2018 Oct 4;13(10):e0205038. doi: 10.1371/journal.pone.0205038 (PMC6171917; doi:10.1371/journal.pone.0205038)

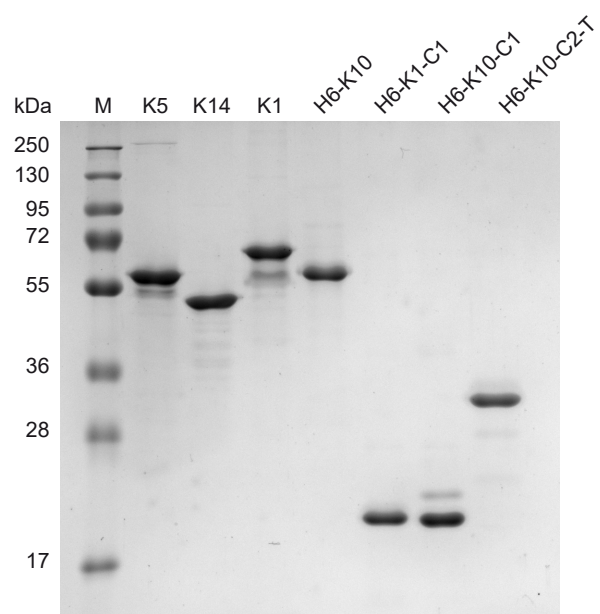

Supplement: S2 Fig — The indicated proteins (1 μg/lane) were size fractionated on 12% SDS-PAGE that was stained with Coomassie brilliant blue. M, markers. (PDF) [file pone.0205038.s002.pdf]

A

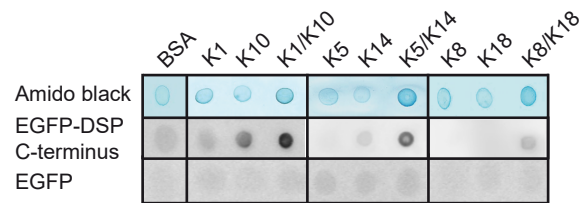

B

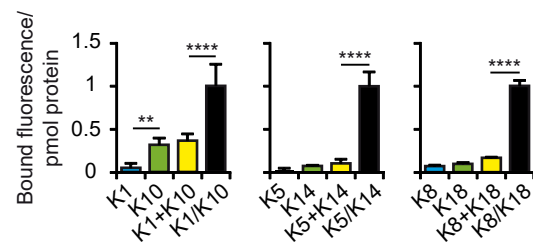

Supplement: S3 Fig — A) Nitrocellulose membranes, spotted with BSA (control) and individual or mixed (/) cytokeratins (3 pmol/spot) were stained with amido black or overlaid with soluble extracts of HEK 293T cells expressing either EGFP-DSP C-terminus (55 ± 21 nM, total concentration) or EGFP (≥ 60 nM) and scanned for fluorescence. B) Quantified fluorescence signals are relative to the normalized results obtained with each mixture (/) of keratins (K1/K10, K5/K14 and K8/K18). K1 + K10, K5 + K14 and K8 + K18 correspond to the sum of the fluorescence signals obtained with the individual proteins (not mixed). Mean ± SD, n≥3; ANOVA-Tukey test; ** and ****, residual P < 0.01 and < 0.0001, respectively. (PDF) [file pone.0205038.s003.pdf]

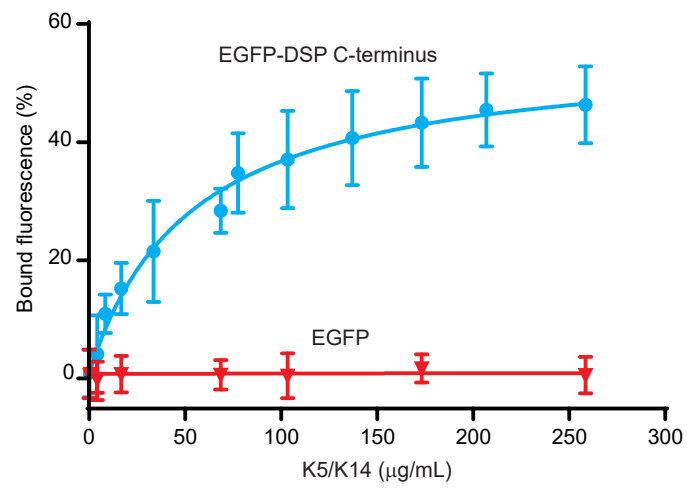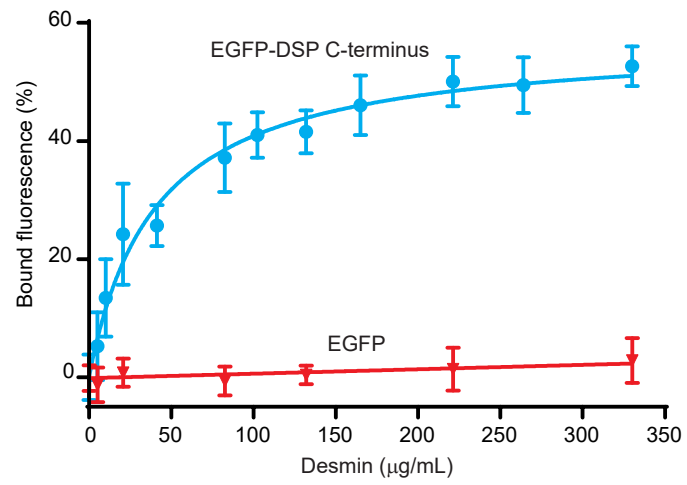

Supplement: S4 Fig — Variable concentrations of K5/K14 IFs or vimentin IFs, preassembled in vitro, were mixed with a constant concentration (22 nM) of EGFP-DSP C-terminus or EGFP in the soluble extracts of HEK 293T cells. Representative binding curves from ≥ 3 independent experiments are shown. (PDF) [file pone.0205038.s004.pdf]
