# Supplementary material for: Desmoplakin interacts with the coil 1 of different types of intermediate filament proteins and displays high affinity for assembled intermediate filaments
Source: PLoS One. 2018 Oct 4;13(10):e0205038. doi: 10.1371/journal.pone.0205038 (PMC6171917; doi:10.1371/journal.pone.0205038)
Supplement: S5 Fig — The MAFFT algorithm was used; *,:, and. denote identity, conserved and semi-conserved amino acid substitutions, respectively. The position in the protein sequence of the first amino acid in each row is indicated. (PDF) [file pone.0205038.s005.pdf]

```
                                 Coil 1A                             Coil 1B
                   __________________________________                ________
K1             181 REQIKSLNNQFASFIDKVRFLEQQNQVLQTKWELLQQVDTST----RTHNLEPYFESFIN
K3             199 REQIKTLNNKFASFIDKVRFLEQQNKVLETKWNLLQQQGTSSIS--GTNNLEPLFENHIN
K5             169 REQIKTLNNKFASFIDKVRFLEQQNKVLDTKWTLLQEQGTKT----VRQNLEPLFEQYIN
K6A            164 REQIKTLNNKFASFIDKVRFLEQQNKVLETKWTLLQEQGTKT----VRQNLEPLFEQYIN
K6B            164 REQIKTLNNKFASFIDKVRFLEQQNKVLDTKWTLLQEQGTKT----VRQNLEPLFEQYIN
K6C            164 REQIKTLNNKFASFIDKVRFLEQQNKVLDTKWTLLQEQGTKT----VRQNLEPLFEQYIN
K75            150 REQIKTLNNKFASFIDKVRFLEQQNKVLETKWALLQEQGSRT----VRQNLEPLFDSYTS
K4             138 REQIKLLNNKFASFIDKVQFLEQQNKVLETKWNLLQQQTTTT----SSKNLEPLFETYLS
K76            184 REQIKTLNNKFASFIDKVRFLEQQNKVLETKWELLQQQTTGS----GPSSLEPCFESYIS
K79            143 REQIKTLNNKFASFIDKVRFLEQQNKVLETKWALLQEQGQNLGV--TRNNLEPLFEAYLG
K71            131 REQIKALNNKFASFIDKVRFLEQQNQVLETKWELLQQLDLNN----CKNNLEPILEGYIS
K73            133 REQIKVLNNKFASFIDKVRFLEQQNQVLETKWELLQQLDLNN----CKNNLEPILEGYIS
K74            141 REQIKVLNDKFASFIDKVRFLEQQNQVLETKWELLQQLDLNN----CKKNLEPILEGYIS
K72            126 REQIKALNNKFASFIDKVRFLEQQNQVLETKWNLLQQLDLNN----CRKNLEPIYEGYIS
K77            165 REQIMVLNNKFASFIDKVRFLEQQNQVLQTKWELLQQVNTST----GTNNLEPLLENYIG
K2             179 REQIKTLNNKFASFIDKVRFLEQQNQVLQTKWELLQQMNVGT----RPINLEPIFQGYID
K7              92 SEQIKTLNNKFASFIDKVRFLEQQNKLLETKWTLLQEQKSAK-----SSRLPDIFEAQIA
K8              92 KEQIKTLNNKFASFIDKVRFLEQQNKMLETKWSLLQQQKTAR------SNMDNMFESYIN
K78            112 TQEIRTLNNQFASFIDKVRFLEQQNKVLETKWHLLQQQGLSG----SQQGLEPVFEACLD
K80             84 KEEMKALNDKFASLIGKVQALEQRNQLLETRWSFLQGQDS------AIFDLGHLYEEYQG
Desmin         109 KVELQELNDRFANYIEKVRFLEQQNAALAAEVNRLKGREP--------TRVAELYEEELR
Vimentin       104 KVELQELNDRFANYIDKVRFLEQQNKILLAELEQLKGQGK--------SRLGDLYEEEMR
K9             154 KSTMQELNSRLASYLDKVQALEEANNDLENKIQDWYDKKGPA----AIQKNYSPYYNTID
K10            147 KVTMQNLNDRLASYLDKVRALEESNYELEGKIKEWYEKHGNSHQ--GEPRDYSKYYKTID
K13            105 KITMQNLNDRLASYLEKVRALEEANADLEVKIRDWHLKQSPA----SPERDYSPYYKTIE
K15            106 KITMQNLNDRLASYLDKVRALEEANADLEVKIHDWYQKQTPT----SPECDYSQYFKTIE
K14            116 KVTMQNLNDRLASYLDKVRALEEANADLEVKIRDWYQRQRP-----AEIKDYSPYFKTIE
K16            118 KVTMQNLNDRLASYLDKVRALEEANADLEVKIRDWYQRQRP-----SEIKDYSPYFKTIE
K17             85 KATMQNLNDRLASYLDKVRALEEANTELEVKIRDWYQRQAP-----GPARDYSQYYRTIE
K19             81 KLTMQNLNDRLASYLDKVRALEAANGELEVKIRDWYQKQGP-----GPSRDYSHYYTTIQ
K12            126 KETMQNLNDRLASYLDKVRALEEANTELENKIREWYETRGTGTAD-ASQSDYSKYYPLIE
K24            141 KQTMQNLNDRLANYLDKVRALEEANTDLENKIKEWYDKYGPGSGDGGSGRDYSKYYSIIE
K25             80 KVTMQNLNDRLASYLDSVHALEEANADLEQKIKGWYEKFGPGSCR-GLDHDYSRYFPIID
K27             85 KVTMQNLNDRLASYLENVRALEEANADLEQKIKGWYEKFGPGSCR-GLDHDYSRYFPIID
K28             87 KVTMQNLNDRLASYLDNVRALEEANAELERKIKGWYEKYGPGSCR-GLDHDYSRYHLTIE
K26             84 KVTMQNLNDRLASYLDHVHALEEANADLEQKIKGWYEKCEPGSSR-EHDHDYSRYFSVIE
K18             81 KETMQSLNDRLASYLDRVRSLETENRRLESKIREHLEKKGP------QVRDWSHYFKIIE
K20             71 KMAMQNLNDRLASYLEKVRTLEQSNSKLEVQIKQWYETNAP-----RAGRDYSAYYRQIE
K23             73 KATMQNLNDRLASYLEKVRALEEANMKLESRILKWHQQRDP-----GSKKDYSQYEENIT
                      :  **.::*. :  *: **  *  * .
```

Coil 1B

```
                    ____________________________________________________________
K1              237 NLRRRVDQLKSDQSRLDSELKNMQDMVEDYRNKYEDEINKRTNAENEFVTIKKDVDGAYM
K3              257 YLRSYLDNILGERGRLDSELKNMEDLVEDFKKKYEDEINKRTAAENEFVTLKKDVDSAYM
K5              225 NLRRQLDSIVGERGRLDSELRNMQDLVEDFKNKYEDEINKRTTAENEFVMLKKDVDAAYM
K6A             220 NLRRQLDSIVGERGRLDSELRGMQDLVEDFKNKYEDEINKRTAAENEFVTLKKDVDAAYM
K6B             220 NLRRQLDNIVGERGRLDSELRNMQDLVEDLKNKYEDEINKRTAAENEFVTLKKDVDAAYM
K6C             220 NLRRQLDSIVGERGRLDSELRNMQDLVEDLKNKYEDEINKRTAAENEFVTLKKDVDAAYM
K75             206 ELRRQLESITTERGRLEAELRNMQDVVEDFKVRYEDEINKRTAAENEFVALKKDVDAAYM
K4              194 VLRKQLDTLGNDKGRLQSELKTMQDSVEDFKTKYEEEINKRTAAENDFVVLKKDVDAAYL
K76             240 FLCKQLDSLLGERGNLEGELKSMQDLVEDFKKKYEDEINKRTAAENEFVGLKKDVDAAFM
K79             201 SMRSTLDRLQSERGRLDSELRNVQDLVEDFKNKYEDEINKHTAAENEFVVLKKDVDAAYM
K71             187 NLRKQLETLSGDRVRLDSELRNVRDVVEDYKKRYEEEINKRTAAENEFVLLKKDVDAAYA
K73             189 NLRKQLETLSGDRVRLDSELRSVREVVEDYKKRYEEEINKRTTAENEFVVLKKDVDAAYT
K74             197 NLRKQLETLSGDRVRLDSELRSMRDLVEDYKKRYEVEINRRTTAENEFVVLKKDADAAYA
K72             182 NLQKQLEMLSGDGVRLDSELRNMQDLVEDYKKRYEVEINRRTAAENEFVVLKKDVDAAYM
K77             221 DLRRQVDLLSAEQMRQNAEVRSMQDVVEDYKSKYEDEINKRTGSENDFVVLKKDVDAAYV
K2              235 SLKRYLDGLTAERTSQNSELNNMQDLVEDYKKKYEDEINKRTAAENDFVTLKKDVDNAYM
K7              147 GLRGQLEALQVDGGRLEAELRSMQDVVEDFKNKYEDEINHRTAAENEFVVLKKDVDAAYM
K8              146 NLRRQLETLGQEKLKLEAELGNMQGLVEDFKNKYEDEINKRTEMENEFVLIKKDVDEAYM
K78             168 QLRKQLEQLQGERGALDAELKACRDQEEEYKSKYEEEAHRRATLENDFVVLKKDVDGVFL
K80             138 RLQEELRKVSQERGQLEANLLQVLEKVEEFRIRYEDEISKRTDMEFTFVQLKKDLDAECL
Desmin          161 ELRRQVEVLTNQRARVDVERDNLLDDLQRLKAKLQEEIQLKEEAENNLAAFRADVDAATL
Vimentin        156 ELRRQVDQLTNDKARVEVERDNLAEDIMRLREKLQEEMLQREEAENTLQSFRQDVDNASL
K9              210 DLKDQIVDLTVGNNKTLLDIDNTRMTLDDFRIKFEMEQNLRQGVDADINGLRQVLDNLTM
K10             205 DLKNQILNLTTDNANILLQIDNARLAADDFRLKYENEVALRQSVEADINGLRRVLDELTL
K13             161 ELRDKILTATIENNRVILEIDNARLAADDFRLKYENELALRQSVEADINGLRRVLDELTL
K15             162 ELRDKIMATTIDNSRVILEIDNARLAADDFRLKYENELALRQGVEADINGLRRVLDELTL
K14             171 DLRNKILTATVDNANVLLQIDNARLAADDFRTKYETELNLRMSVEADINGLRRVLDELTL
K16             173 DLRNKIIAATIENAQPILQIDNARLAADDFRTKYEHELALRQTVEADVNGLRRVLDELTL
K17             140 ELQNKILTATVDNANILLQIDNARLAADDFRTKFETEQALRLSVEADINGLRRVLDELTL
K19             136 DLRDKILGATIENSRIVLQIDNARLAADDFRTKFETEQALRMSVEADINGLRRVLDELTL
K12             185 DLRNKIISASIGNAQLLLQIDNARLAAEDFRMKYENELALRQGVEADINGLRRVLDELTL
K24             201 DLRNQIIAATVENAGIILHIDNARLAADDFRLKYENELCLRQSVEADINGLRKVLDDLTM
K25             139 DLKNQIIASTTSNANAVLQIDNARLTADDFRLKYENELALHQSVEADVNGLRRVLDEITL
K27             144 ELKNQIISATTSNAHVVLQNDNARLTADDFRLKFENELALHQSVEADINGLRRVLDELTL
K28             146 DLKNKIISSTTTNANVILQIDNARLAADDFRLKYENELTLHQNVEADINGLRRVLDELTL
K26             143 DLKRQIISATICNASIVLQNDNARLTADDFRLKYENELALHHSVEADTSGLRRVLDELTL
K18             135 DLRAQIFANTVDNARIVLQIDNARLAADDFRVKYETELAMRQSVENDIHGLRKVIDDTNI
K20             126 ELRSQIKDAQLQNARCVLQIDNAKLAAEDFRLKYETERGIRLTVEADLQGLNKVFDDLTL
K23             128 HLQEQIVDGKMTNAQIILLIDNARMAVDDFNLKYENEHSFKKDLEIEVEGLRRTLDNLTI
                     :   :                        . : : *   :   :     :.   *
```

Coil 1B

```
K1            297 TKVDLQAKLDNLQQEIDFLTALYQAELSQMQT
K3            317 NKVELQAKVDALIDEIDFLRTLYDAELSQMQS
K5            285 NKVELEAKVDALMDEINFMKMFFDAELSQMQT
K6A           280 NKVELQAKADTLTDEINFLRALYDAELSQMQT
K6B           280 NKVELQAKADTLTDEINFLRALYDAELSQMQT
K6C           280 NKVELQAKADTLTDEINFLRALYDAELSQMQT
K75           266 NKVELEAKVKSLPEEINFIHSVFDAELSQLQT
K4            254 NKVELEAKVDSLNDEINFLKVLYDAELSQMQT
K76           300 NKVELQAKVDSLTDEVSFLRTLYEMELSQMQS
K79           261 GRMDLHGKVGTLTQEIDFLQQLYEMELSQVQT
K71           247 NKVELQAKVESMDQEIKFFRCLFEAEITQIQS
K73           249 SKVELQAKVDALDGEIKFFKCLYEGETAQIQS
K74           257 VKVELQAKVDSLDKEIKFLKCLYDAEIAQIQT
K72           242 NKVELQAKVDSLTDEIKFFKCLYEGEITQIQS
K77           281 SKVDLESRVDTLTGEVNFLKYLFLTELSQVQT
K2            295 IKVELQSKVDLLNQEIEFLKVLYDAEISQIHQ
K7            207 SKVELEAKVDALNDEINFLRTLNETELTELQS
K8            206 NKVELESRLEGLTDEINFLRQLYEEEIRELQS
K78           228 SKMELEGKLEALREYLYFLKHLNEEELGQLQT
K80           198 HRTELETKLKSLESFVELMKTIYEQELKDLAA
Desmin        221 ARIDLERRIESLNEEIAFLKKVHEEEIRELQA
Vimentin      216 ARLDLERKVESLQEEIAFLKKLHEEEIQELQA
K9            270 EKSDLEMQYETLQEELMALKKNHKEEMSQLTG
K10           265 TKADLEMQIESLTEELAYLKKNHEEEMKDLRN
K13           221 SKTDLEMQIESLNEELAYMKKNHEEEMKEFSN
K15           222 ARTDLEMQIEGLNEELAYLKKNHEEEMKEFSS
K14           231 ARADLEMQIESLKEELAYLKKNHEEEMNALRG
K16           233 ARTDLEMQIEGLKEELAYLRKNHEEEMLALRG
K17           200 ARADLEMQIENLKEELAYLKKNHEEEMNALRG
K19           196 ARTDLEMQIEGLKEELAYLKKNHEEEISTLRG
K12           245 TRTDLEMQIESLNEELAYMKKNHEDELQSFRV
K24           261 TRSDLEMQIESFTEELAYLRKNHEEEMKNMQG
K25           199 CRTDLEIQYETLSEEMTYLKKNHKEEMQVLQC
K27           204 CRTDLEIQLETLSEELAYLKKNHEEEMKALQC
K28           206 CRTDQELQYESLSEEMTYLKKNHEEEMKALQC
K26           203 CTTDLEIQCETLSEELTYLKKSHEEEMEVLQY
K18           195 TRLQLETEIEALKEELLFMKKNHEEEVKGLQA
K20           186 HKTDLEIQIEELNKDLALLKKEHQEEVDGLHK
K23           188 VTTDLEQEVEGMRKELILMKKHHEQEMEKHHV
                     : . .   :   :  :      *
```
