# Supplementary material for: Desmoplakin interacts with the coil 1 of different types of intermediate filament proteins and displays high affinity for assembled intermediate filaments
Source: PLoS One. 2018 Oct 4;13(10):e0205038. doi: 10.1371/journal.pone.0205038 (PMC6171917; doi:10.1371/journal.pone.0205038)
Supplement: S1 Table — (PDF) [file pone.0205038.s006.pdf]

S1 Table. Tested recombinant IF proteins.

Recombinant protein[1)]	Domain	Species	Amino acid first	last
K1	full length	human	1	644
K1-C1	coil 1	mouse	189	336
K5	full length	human	1	540
K5-C1	coil 1	human	169	316
K5-C2-T	linker 12-tail	human	317	590
K8	full length	human	1	483
K10	full length	human	1	584
K10-C1	coil 1	mouse	136	285
K10-C2-T	linker 12-tail	human	296	584
K14	full length	human	1	472
K18	full length	human	1	430
desmin	full length	human	1	470
desmin-rod	rod	human	105	416
desmin-C1	coil 1-linker 12	human	94	274
desmin-C2	coil 2	human	269	416
vimentin	full length	human	1	466
vimentin-rod	rod	human	80	413
vimentin-C1	coil 1-linker 12	human	80	268
vimentin-C2	linker 12-coil 2	human	247	415

[1)] Genbank accession No: K1, NP_006112; mouse K1, NP_032499; K5, NP_000415; K8, NP_001243222; K10, NP_000412; mouse K10, NP_034790; K14, NP_000517; K18, NP_000215; desmin, NP_001918; and vimentin, NP_00337.
