# Supplementary material for: Desmoplakin interacts with the coil 1 of different types of intermediate filament proteins and displays high affinity for assembled intermediate filaments
Source: PLoS One. 2018 Oct 4;13(10):e0205038. doi: 10.1371/journal.pone.0205038 (PMC6171917; doi:10.1371/journal.pone.0205038)
Supplement: S2 Table — (PDF) [file pone.0205038.s007.pdf]

S2 Table. Y2H assay results with some K5 and K14 constructs used to map the interacting domain(s) with the C-terminus of desmoplakin in Y3H assays.

pAS2.1 vector			pACT2 vector			Y2H results	
GAL4-DNA-BD fusion protein	Amino acid no	Domain(s)	GAL4-AD fusion protein	Amino acid no	Domain(s)	-His[1)]	-Ade
K5	1-590	all	K14	1-472	all	+	+
K5	1-590	all	K14	1-278	head-rod 1-linker 12	+	+
K5	1-590	all	K14	263-472	linker 12-rod 2-tail	+	+
K5	1-333	head-rod 1-linker 12	K14	1-472	all	+	+
K5	317-590	linker 12-rod 2-tail	K14	1-472	all	+	+
K5	1-333	head-rod 1-linker 12	K14	1-278	head-rod 1-linker 12	+	+

[1)] + indicates growth on selection medium without histidine (His) or adenine (Ade). None of the tested keratin construct sustained yeast growth in the absence of a keratin partner in the opposite plasmid (empty vector).
