# Supplementary material for: Desmoplakin interacts with the coil 1 of different types of intermediate filament proteins and displays high affinity for assembled intermediate filaments
Source: PLoS One. 2018 Oct 4;13(10):e0205038. doi: 10.1371/journal.pone.0205038 (PMC6171917; doi:10.1371/journal.pone.0205038)
Supplement: S3 Table — (PDF) [file pone.0205038.s008.pdf]

S3 Table. *KRT5* and *KRT14* variants associated with EBS and tested in Y3H assays with their wild-type partner and the C-terminus of desmoplakin.

					Y3H results	
**Protein**	**Variant[1)]**	**Domain**	**Y3H partners**		**-His**	**-Ade**
K5	p.(Glu168Asp)	head	K14	DSP C-terminus	+	+
	p.(Glu168Gln)	head	K14	DSP C-terminus	+	+
	p.(Asn176Ser)	coil 1A	K14	DSP C-terminus	+	+
	p.(Asn177Ser)	coil 1A	K14	DSP C-terminus	+	+
	p.(Leu311Arg)	coil 1B	K14	DSP C-terminus	+	+
K14	p.(Arg125His)	coil 1A	K5	DSP C-terminus	+	+
	p.(Ile176Met)	coil 1B	K5	DSP C-terminus	+	+
	p.(Val270Met)	linker 12	K5	DSP C-terminus	+	+
	p.(Leu284Pro)	coil 2A	K5	DSP C-terminus	+	+

[1)] https://www.ncbi.nlm.nih.gov/clinvar, *KRT5* or *KRT14*
