# Supplementary material for: Desmoplakin interacts with the coil 1 of different types of intermediate filament proteins and displays high affinity for assembled intermediate filaments
Source: PLoS One. 2018 Oct 4;13(10):e0205038. doi: 10.1371/journal.pone.0205038 (PMC6171917; doi:10.1371/journal.pone.0205038)
Supplement: S4 Table — (PDF) [file pone.0205038.s009.pdf]

S4 Table. Apparent Kd of plakins for IF proteins reported in the literature.

Plakin		IF					
**Protein**	**Domain(s)**	**Protein(s)**	**Domain**	**Type of binding assay**	**Immobilized protein(s)**	**Apparent Kd (mM)**	**Reference**
envoplakin	PRD	vimentin	monomeric coil 1	surface plasmon resonance	IF	19	[1]
periplakin	truncated rod-linker	K8/K18	full length/ filaments	co-sedimentation	n. a.[1)]	2	[2]
plectin	PRD 5-linker	vimentin	full length/ filaments	overlay/Eu^{3+}-labeled IF	plakin	0.05-0.15	[3, 4]
plectin	PRD 5-linker	K5/K14	full length/ filaments	overlay/ EGFP-plakin	IFs	0.1-0.14	[5]
plectin	PRD 5 to PRD 6	K5/K14	full length/ filaments	overlay/ EGFP-plakin	IFs	0.06	[5]
plectin	PRD 5 to C-terminus	K5/K14	full length/ filaments	overlay/ EGFP-plakin	IFs	0.05	[5]

1) n. a., not applicable.

References

1. Fogl C, Mohammed F, Al-Jassar C, Jeeves M, Knowles TJ, Rodriguez-Zamora P, et al. Mechanism of intermediate filament recognition by plakin repeat domains revealed by envoplakin targeting of vimentin. Nat Commun. 2016;7:10827. doi: 10.1038/ncomms10827. PubMed PMID: 26935805.

2. Kalinin AE, Kalinin AE, Aho M, Uitto J, Aho S. Breaking the connection: caspase 6 disconnects intermediate filament-binding domain of periplakin from its actin-binding N-terminal region. J Invest Dermatol. 2005;124(1):46-55. doi: 10.1111/j.0022-202X.2004.23507.x. PubMed PMID: 15654952.
3. Nikolic B, Mac Nulty E, Mir B, Wiche G. Basic amino acid residue cluster within nuclear targeting sequence motif is essential for cytoplasmic plectin-vimentin network junctions. J Cell Biol. 1996;134(6):1455-67. Epub 1996/09/01. PubMed PMID: 8830774; PubMed Central PMCID: PMC2121005.
4. Spurny R, Abdoulrahman K, Janda L, Runzler D, Kohler G, Castanon MJ, et al. Oxidation and nitrosylation of cysteines proximal to the intermediate filament (IF)-binding site of plectin: effects on structure and vimentin binding and involvement in IF collapse. J Biol Chem. 2007;282(11):8175-87. Epub 2007/01/17. doi: 10.1074/jbc.M608473200. PubMed PMID: 17224453.
5. Bouameur JE, Favre B, Fontao L, Lingasamy P, Begre N, Borradori L. Interaction of plectin with keratins 5 and 14: dependence on several plectin domains and keratin quaternary structure. J Invest Dermatol. 2014;134(11):2776-83. doi: 10.1038/jid.2014.255. PubMed PMID: 24940650.
